# Clinical characteristics of patients with diabetic peripheral neuropathy: a retrospective descriptive study

**DOI:** 10.3389/fcdhc.2026.1792894

**Published:** 2026-04-15

**Authors:** Dana Rashwan, Bashair M. Mussa, Amena Sadiya, Rawoof Khan, Salah Abusnana

**Affiliations:** 1 Basic Medical Science Department, College of Medicine, University of Sharjah, Sharjah, United Arab Emirates; 2 Clinical Science Department, College of Medicine, University of Sharjah, Sharjah, United Arab Emirates; 3 Diabetes and Endocrinology Department, University Hospital Sharjah, Sharjah, United Arab Emirates

**Keywords:** diabetic peripheral neuropathy, foot ulcers, risk factors, sensory loss, type 2 diabetes, UAE

## Abstract

**Background:**

Diabetic Peripheral Neuropathy (DPN) is a common complication of diabetes, particularly in regions with high disease prevalence, such as the United Arab Emirates (UAE). This study aims to describe the clinical characteristics of patients with DPN and compare differences in patient profiles according to the status of protective foot sensation.

**Methods:**

This retrospective cross-sectional study included 387 patients with Type 2 diabetes diagnosed with DPN at University Hospital Sharjah (UHS) between 2019 and 2024. Data were extracted from medical records. Patients were grouped by protective sensation status and compared using t-tests, chi-square tests, and logistic regression.

**Results:**

Among the cohort, 45% had Loss of Protective sensation(LOPS) and 55% had reduced protective sensation (RPS). Significant differences were observed in smoking status, active ulcers, amputation, Chronic Kidney Disease severity, lipid profile, and HbA1c levels. Multivariable regression analysis identified smoking (OR = 38.91), ulcer history (OR = 3.35), amputation (OR = 3.46), and severe CKD (OR = 6.04) as predictors of LOPS (all p<0.01).

**Conclusion:**

This study confirms prior findings on DPN and highlights new associations related to loss of protective sensation. Patients with LOPS had higher rates of smoking, renal dysfunction, dyslipidemia, foot complications, and osteomyelitis. Smoking, ulcer or amputation history, and advanced CKD were significantly associated with LOPS. These findings emphasize the need for early detection and targeted interventions to prevent DPN progression.

## 1 Introduction

### 1.1 Background and aims

Diabetes mellitus (DM) remains a major global health challenge, affecting approximately 425 million individuals worldwide, with projections reaching 629 million by 2045 (1). The burden of type 2 diabetes mellitus (T2DM) is particularly high in the United Arab Emirates (UAE), where prevalence is expected to reach 21.4% by 2030 (2). Diabetic peripheral neuropathy (DPN) is the most common microvascular complication of T2DM, affecting approximately 34–35% of individuals with diabetes in the UAE.

Individuals with DPN frequently present with neuropathic symptoms such as burning pain, electric shock–like sensations, paresthesia, diminished ankle reflexes, and sensory numbness (3). On clinical examination, impaired perception of light touch, vibration, and temperature represents the most common objective finding in the diagnosis of DPN (4). Suboptimal management of DPN may lead to serious complications, including foot ulceration, secondary infections, and, in severe cases, lower-limb amputation (4). DPN substantially impairs quality of life, contributing to chronic pain, increased risk of falls, foot ulceration, and potential lower-limb amputation (5).

Multiple diagnostic tools are available for the screening and diagnosis of DPN, including quantitative sensory testing (QST), validated clinical scoring instruments, and electrophysiological assessments such as electromyography (EMG) and nerve conduction studies (NCS). Among these, NCS is considered the gold, particularly in research settings, due to its objective evaluation of large-fiber nerve function. However, its use in clinical practice remains limited due to its time-consuming nature and high cost (6). In addition, the Weinstein 10 g monofilament and 128-Hz tuning fork are widely used as a simple bedside tools in clinical practice in the UAE for clinical assessment (7). Various studies have reported the effectiveness of monofilament testing in screening DPN. It has been reported as very efficient for clinical use. The Weinstein monofilament exerts a pressure of 10 g which should be detected by any normal person to rule out the presence of DPN and it has been used to date with high success. However, there could be certain concerns for its sensitivity due to differences in demographic, social, ethnic, religious, and occupational characteristics of the diabetic person (8).

The management of diabetes becomes increasingly complex once neuropathy has developed. Therefore, routine clinical screening for the severity of loss of protective sensation (LOPS) and early identification of risk factors associated with progressive neuropathy are essential. Timely detection facilitates targeted interventions, optimizes glycemic and risk-factor control, and reduces the likelihood of severe DPN-related complications.

While its debilitating impacts of DPN are relatively well documented, far less is known about the prevalence of the severity of DPN and its risk factors among individuals in this region.

with diabetes. As evidence indicates that risk factors for the progression of DPN include the duration of diabetes, age, HbA1c, Diabetic retinopathy, smoking, BMI, fasting plasma glucose, blood urea nitrogen, and diastolic blood pressure (9). Thus, early clinical detection and risk stratification are essential. In the UAE, literature addressing the clinical characteristics of patients with severe DPN particularly those with LOPS and risk factors associated with severity of symptoms is limited and reporting this data could be beneficial in adding value to clinical management of DPN in the region. Given the lack of specific treatments targeting the underlying pathophysiology of DPN and the predominant focus on symptom management, early detection and intervention are paramount. Clinicians must be well-versed in the clinical features of these patients with absent sensation to promptly identify DPN and prevent associated complications. However, in the UAE, there is a significant deficiency in the literature concerning this topic. To address this deficiency, the present study seeks to describe the clinical characteristics observed among patients with DPN, with a particular focus on their baseline data, foot-related characteristics, comorbidities, and biochemical test profiles. Furthermore, we also aim to compare patients’ clinical features based on their level of loss in protective sensation and investigate factors associated with absent sensation compared to reduced sensation. This comprehensive approach will not only enhance our understanding of severe DPN but also contribute valuable insights for improving patient care and management strategies in clinical settings.

This study aims to characterize the clinical characteristics of patients with DPN at a tertiary care hospital in the United Arab Emirates. Additionally, it seeks to compare clinical characteristics between patients with Reduced protective sensation (RPS) and Loss of protective sensation (LOPS) to identify independent risk factors.

## 2 Methods

### 2.1 Study design

This retrospective cross-sectional study was conducted at the University Hospital Sharjah (UHS), UAE. The study included adult patients with T2DM diagnosed with DPN based on foot examination between January 1, 2019, and January 1, 2024. An non-probability convenience sampling method was used to collect data from all patients diagnosed with DPN.

### 2.3 Sample size calculation

Based on standard sample size estimation formulas and accounting for an expected 10% exclusion rate, the minimum required sample size was calculated as 382. A total of 387 complete patient records were ultimately included. Oversampling enhances generalizability and minimizes the effect of missing or incomplete data on study outcomes.

### 2.4 Ethical approval

The ethical approval was granted by UHS Research and Ethics Committee (Ref. No: UHS-HERC-133-05062023). As the study was retrospective in nature, written informed consent was waived. All patient data were anonymized and coded to ensure confidentiality. Only the principal investigator, supervisor, co-supervisor, and statistician had access to the dataset.

### 2.5 Data collection procedure

Adults (≥18 years) with T2DM from all nationalities who underwent foot examination and were diagnosed with DPN using the 10g monofilament were included in the study. Results were categorized into two groups: Reduced Protective sensation (RPS) (1 to 7/10 sites felt), or LOPS (0/10 sites felt). Since the inclusion criteria included patient diagnosed with DPN, the patients reporting normal sensation were excluded. strengthen use of monofilament with studies.

#### 2.5.1 Data collection process

The data collection process was conducted from November 2023 to February 2024. After the ethical approval, the patient list was generated using the hospital live system software.

The initial search yielded 2970 patients, which was refined to 500 eligible entries after removing duplicates and non-relevant diagnosis. After thorough review, 399 patients met diagnostic criteria for DPN; 12 more were excluded due to missing data, resulting in a final sample of 387 patients. Demographic data and clinical data was collected along with symptoms typical of diabetic neuropathy, such as pins and needles, tingling, numbness, and shooting pain in the feet.

The biochemical parameters results includes HbA1c, estimated glomerular filtration rate (eGFR), serum creatinine, urea plasma, and lipid profile (HDL-c, LDL-c, triglycerides, and total cholesterol) were obtained. Medications prescribed to manage diabetes mellitus, including oral tablets, insulin therapy, injectables, or a combination, were documented. Additionally, any medications prescribed to alleviate symptoms of DPN, such as antidepressants, anticonvulsants, or topical agents, were also recorded. standardize term ‘absent sensation’ or loss of protective sensation, LOPS.

### 2.6 Statistical analysis

Data were analyzed using SPSS version 29.0 (SPSS, Chicago, IL, USA). Descriptive analysis was performed, producing means and standard deviation (SD) for continuous data, and counts and frequencies for categorical variables. The Chi-square test was utilized to compare proportions for categorical data, while independent t-tests were employed to examine differences in means for continuous data. Logistic regression including Odds ratio (OR) and 95% confidence interval (CI) were calculated to determine the factors associated with LOPS as compared to RPS. The selection of confounding variables for adjustment in OR calculations, such as gender, BMI, glycemic control, and diabetes duration was based on reporting of previous literature. Statistical significance was defined as a p-value of <0.05, using a two-sided p-value. Collinearity among independent variables was assessed using the Variance Inflation Factor (VIF) in IBM SPSS Statistics, with a VIF value of less than 5 considered indicative of no significant multicollinearity. In addition, all statistical analyses were reviewed and verified by a professional statistician to ensure the accuracy and robustness of the applied methods.

## 3 Results

### 3.1 Baseline characteristics of the study population

A total of 387 medical records of T2DM patients with peripheral neuropathy were reviewed. The baseline characteristics of DPN patients in the study population are outlined in **Table 1**. As shown in **Table 1**, 92.5% were UAE nationals and 85.5% were aged ≥60 years. The gender distribution was nearly equal (50.9% male, 49.1% female). The proportion of individuals who smoke compared to those who do not smoke was nearly identical (50.4% vs 49.6%). Furthermore, 51.4% of the patients were classified as obese, 33.3% fell into the overweight category, and the remaining 15.2% were categorized as normal weight.

**Table 1:** Baseline characteristics the study population (n= 387).

Variable	n (%) or mean ± SD
Nationality	
Emirati	358(92.5)
Non-Emirati	29(7.5)
Age (years)	
<60	56 (14.5)
≥60	331(85.5)
Gender	
Male	197 (50.9)
Female	190 (49.1)
Diabetes duration (years)	
<10	32 (8.3)
≥10	355 (91.7)
Smoking status	
Smoker	195 (50.4)
Non-smoker	192 (49.6)
Weight (kg)	80.3± 17
Height (cm)	161.5± 9.9
BMI (kg/m^2^)	
Normal (18.5-24.9 kg/m^2^)	59 (15.2)
Overweight (25-29.9 kg/m^2^)	129 (33.3)
Obese (≥30kg/m^2^)	199 (51.4)

BMI, body mass index.

### 3.2 Prevalence of Co-morbidities among patients with DPN in the study population

**Figure 1:**
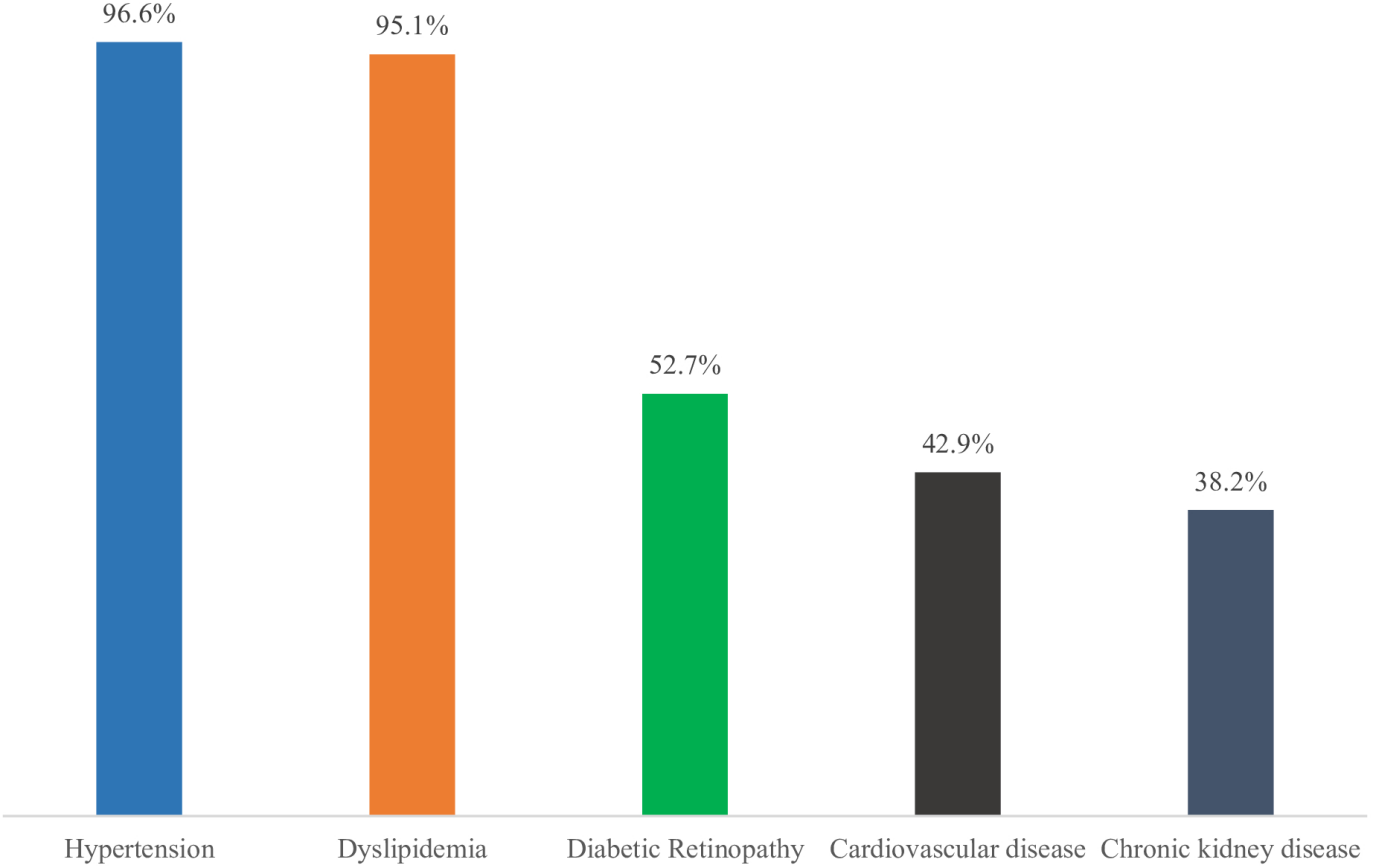
Common co-morbidities observed among the study population. *CVD includes ischemic heart disease and heart failure.

As shown in **Figure 1**, the majority of patients presented with multiple comorbidities. Hypertension (96.6%) and dyslipidemia (95.1%) were the most prevalent. Diabetic retinopathy (DR) affected 52.7% of patients, with 41.1% having non-proliferative (NPDR) and 11.6% proliferative (PDR) forms. Cardiovascular disease (CVD) was present in 42.9%, and chronic kidney disease (CKD) in 38.2%, categorized as mild (7.5%), moderate (23%), or severe (7.7%).

### 3.3 Medications used in the management of diabetes and DPN

**Figure 2:**
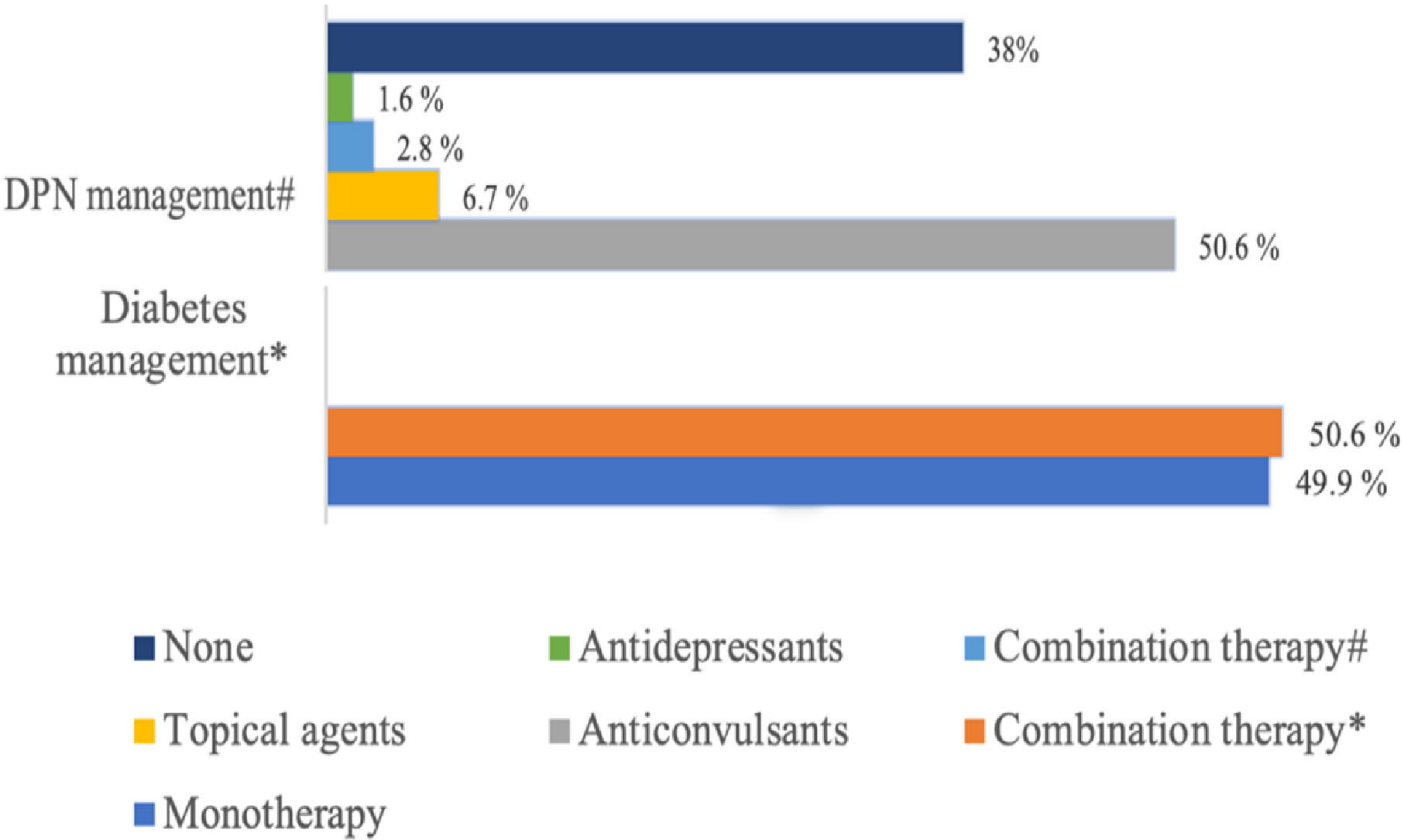
Management of diabetes and DPN among the study population. *Monotherapy includes the use of insulin only or oral medications only. Combination therapy means the use of oral+insulin/oral+injectables or insulin +injectables. #Combination therapy in this context means the use of anticonvulsants + antidepressants. Topical agents include lidocaine or capsaicin cream.

A slightly higher proportion of patients (50.6%) were receiving combination therapy for diabetes management, compared to 49.4% on monotherapy. In terms of DPN management, anticonvulsants were the most commonly prescribed class (50.6%), followed by topical agents (6.7%), combination therapy (2.8%), and antidepressants (1.6%). Notably, 38% of patients were not receiving any pharmacological treatment for DPN, as illustrated in **Figure 2**.

### 3.4 Clinical and biochemical characteristics of patients with DPN

The clinical and biochemical findings is summarized in the **Table 2**. 55% of participants had reduction in protective sensation, whereas 45% of patients experienced a complete absence in sensation. Among the patients, 91.5% reported experiencing symptoms, with 50.4% presenting with positive symptoms, 32.3% exhibiting negative symptoms, and 8.8% reporting both positive and negative symptoms. Clinical examination of the foot showed that 22% of patients had absent pulses, 28% had active ulcers, with 4% of these cases advancing to osteomyelitis. History of ulcers and amputation was noted in 56% and 8% of patients, respectively. Additionally, the majority of patients (71%) were found to be wearing inappropriate footwear, while 22% were using medical or orthopedic footwear. The majority of patients, constituting 86%, did not have Peripheral Vascular Disease (PVD). Finally, vitamin D deficiency was identified in 46% of the patient population. As for the biochemical data, the mean eGFR was 78.7 ml/min, serum creatinine was 101 μmol/l, and urea plasma was 7 mmol/l. The mean HbA1c of the study population was reported to be 8.3%. Additionally, the lipid profile, including HDL-c, LDL-c, total cholesterol, and triglycerides levels were 1.1 mmol/l, 2.4 mmol/l, 3.9 mmol/l, and 1.9 mmol/l respectively.

**Table 2:** Clinical and biochemical characteristics associated with DPN in the study population (n=387).

Variables	N (%) Or Mean± SD
Protective sensation*		
Loss of Protective Sensation Reduced Protective sensation	174(45) 213 (55)
Symptoms^#^		
Positive Negative Both None reported	195 (50.4) 125 (32.3) 34 (8.8) 33 (8.5)
Pedal Pulses**		
Present Absent	303 (78) 84 (22)
Presence of active ulcers		
Yes No	110 (28) 277 (72)
Osteomyelitis		
Yes No	15 (4) 372 (96)
History of ulcers		
Yes No	217 (56) 170 (44)
History of amputation		
Yes No	30 (8) 357 (92)
Footwear		
Suitable (medical/orthopedic) Non-suitable	112 (29) 275 (71)
Peripheral vascular disease		
Yes No	54 (14) 333(86)
Vitamin D deficiency		
Yes No	177 (46) 210 (54)
eGFR (mL/min)	78.7 ± 36.8
Serum creatinine (μmol/L)	101 ± 90.6
Urea plasma (mmol/l)	7 ± 7.5
HbA1c (%)	8.3 ± 2
HDL-c (mmol/L)	1.1 ± 0.34
LDL-c (mmol/L)	2.4 ± 1.1
Total cholesterol (mmol/L)	3.9 ± 1.3
Triglycerides (mmol/L)	1.9 ± 1.2

Egfr, estimated glomerular filtration rate; HbA1c, glycosylated haemoglobin; HDL-c, high-density lipoprotein cholesterol; LDL-c, low-density lipoprotein cholesterol.

*Protective sensation is assessed using 10 g Semmes–Weinstein monofilament evaluation (SWME). LOPS is defined as a complete loss in protective sensation, 0/10 sites felt. Reduced is defined as 1 to 7/10 sites felt.

#Positive symptoms include burning, tingling, pins and needles, and electric shock-like feeling. Negative symptoms include numbness and loss of sensation.

**Pedal pulses were palpated on all 4 sites (left and right: posterior tibial & dorsalis pedis).

Present is defined as palpable pulses on all 4 sites.

Absent is defined as an absent PT or DP pulse in at least one foot.

### 3.5 Comparison of baseline, clinical, and biochemical characteristics between LOPS and RPS

A comparison was undertaken to analyze the characteristics of patients with either LOPS or RPS within the study cohort (**Tables 3**, **4**). The average age of both groups was comparable, with the LOPS group having a mean age of 67.8 years and the RPS group having a mean age of 67.5 years (p=0.795). Patients with LOPS tended to have a longer duration of diabetes, with an average duration of 17.8 years (p=0.053). In the group with LOPS, males comprised the majority (52%), while females were more prevalent in the RPS group (50%) (p=0.771).

**Table 3:** Comparison of baseline and biochemical characteristics of with LOPS vs RPS.

Variables	LOPS (n= 174)	RPS (n= 213)	P value
Age (years), mean ± SD	67.8 ± 10.3	67.5 ± 9.8	0.795
DM duration (years), mean ± SD	17.8 ± 6.8	16.5 ± 6.7	0.053
Gender, n %			
Male Female	90(52) 84(48)	107(50) 106(50)	0.771
Smoking status, n %			
Smokers Non-smokers	156(89.7) 18 (10.3)	39(18.3) 174 (81.7)	<0.001
Height (cm), mean ± SD	162.5± 10.5	160.6± 9.3	0.061
Weight (kg), mean ± SD	80.4± 18.4	80.1 ± 15.9	0.882
BMI (kg/m^2^), n %			
Normal weight Overweight Obese	33 (19) 59 (33.9) 82(47.1)	26 (12.2) 70 (32.9) 117(54.9)	0.133
Creatinine (μmol/l), mean ± SD	123.4 ± 110.5	95.5 ± 68.2	0.003
eGfr (mL/min), mean ± SD	71.7 ± 35.3	84.5 ± 37	<0.001
Urea plasma (mmol/L), mean ± SD	9.6 ± 9.8	7.3 ± 4.7	0.004
LDL-c (mmol/L), mean ± SD	2.5 ± 1.1	2.4 ± 1.2	0.649
Total cholesterol (mmol/L), mean ± SD	3.9 ± 1.2	3.8 ± 1.3	0.357
HbA1c (%), mean ± SD	8.6 ± 2.1	8.1 ± 1.9	0.022
HDL-c (mmol/L), mean ± SD	1 ± 0.34	1.1 ± 0.33	<0.001
Triglycerides (mmol/L), mean ± SD	2.04 ± 1.2	1.7 ± 1.2	0.016

LOPS sensation is defined as complete loss in protective sensation, 0/10 sites felt.

RPS is defined as 1-7/10 sites felt.

**Table 4:** Comparison of clinical characteristics between those with LOPS vs RPS.

Variables	LOPS (n= 174)	RPS (n= 213)	P value
Symptoms, n %				
Positive Negative Both None	81(46.6) 61 (35.1) 13 (7.5) 19 (10.9)	114 (53.5) 64 (30) 21 (9.9) 14 (6.6)	0.221	
Pedal pulses, n %				
Present Absent	136 (78.2) 38 (21.8)	167 (78.4) 46 (21.6)	0.954	
Active ulcer, n %				
Yes No	81 (46.6) 93 (53.4)	29 (13.6) 184 (86.4)	<0.001	
History of ulcers, n %				
Yes No	125 (71.8) 49 (28.2)	92 (43.2) 121 (56.8)	<0.001	
History of amputation, n %				
Yes No	22 (12.6) 152 (87.4)	8 (3.8) 205 (96.2)	0.001	
Foot wear, n %				
Suitable Unsuitable	44 (25.3) 130 (74.7)	68 (31.9) 145 (68.1)	0.152	
Diabetic retinopathy^#^, n %				
NPDR PDR None	72 (41.4) 24 (13.8) 78 (44.8)	87 (40.8) 21 (9.9) 105 (49.3)	0.431	
Dyslipidemia, n %				
Yes No	166 (95.4) 8 (4.6)	202 (94.8) 11 (5.2)	0.797	
Hypertension, n %				
Yes No	170(97.7) 4 (2.3)	204(95.8) 9 (4.2)	0.295	
Chronic kidney disease*, n %				
Mild Moderate Severe None	12 (6.9) 50 (28.7) 16 (9.2) 96 (55.2)	17 (8) 39 (18.3) 14 (6.6) 143 (67.1)	0.005	
Cardiovascular disease, n %				
Yes No	81(46.6) 93 (53.4)	85(40) 128 (60)	0.189	
Vitamin d deficiency, n %				
Yes No	67(38.5) 107 (61.5)	110(51.6) 103 (48.4)	0.010	
Peripheral vascular disease, n %				
Yes No	33(19) 141 (81)	21(10) 192 (90)	0.010	
Osteomyelitis, n %				
Yes No	14(8) 160 (92)	1(0.5) 212 (99.5)	<0.001	

LOPS is defined as complete loss in protective sensation, 0/10 sites felt.

RPS is defined as 1-7/10 sites felt.

*Mild CKD= eGFR; 45 to 89, moderate CKD = eGFR; 30 to 44 and severe CKD= eGFR;<15 to 29.

^#^NPDR, Non-proliferative diabetic retinopathy, PDR, Proliferative diabetic retinopathy.

A significant proportion of patients with LOPS (89.7%) were smokers, in contrast to only 18.3% in the comparison group (p<0.001). Additionally, patients with LOPS tended to be taller, with an average height of 162.5cm (p=0.061), and heavier, with an average weight of 80.4 kg (p=0.882). Conversely, in the reduced sensation group, a higher percentage of patients were classified as obese, at 54.9%, compared to 47.1% in the LOPS group (p=0.133).

Significant differences were noted in the biochemical parameters, with patients exhibiting LOPSshowing more abnormal results. The mean eGFR was notably lower in the LOPS group at 71.7 ml/min compared to 84.5 ml/min in the RPS group (p<0.001). Furthermore, the LOPS group had higher average creatinine levels (123.4 μmol/L, p=0.003) and plasma urea (9.6 mmol/L, p=0.004). HbA1c levels were also elevated in the LOPS group, with a mean of 8.6% compared to 8.1% in the RPS group (p=0.022). As for the lipid profile results, lower HDL-c levels (1 mmol/l, p<0.001) and higher triglyceride levels (2.04 mmol/l, p=0.06) were noted in the LOPS group. Although total cholesterol and LDL-c levels were numerically higher in the LOPS group (2.5 mmol/l and 3.9 mmol/l, respectively), these differences did not reach statistical significance (p=0.357 and p=0.649, respectively).

Furthermore, among the various symptoms exhibited by patients, positive symptoms were the most prevalent in both groups, with a higher rate observed in the RPS group at 53.5%, compared to 46.6% in the LOPS group (p=0.221). Both the LOPS and RPS groups showed a similar proportion of patients with LOPS pedal pulses, approximately 21.8% and 21.6%, respectively (p=0.954). In the LOPS group, 74.7% were found to have inappropriate footwear, slightly higher than the 68.1% observed in the RPS group (p=0.152).

Significant differences were noted between the two groups in terms of the prevalence of active ulcers, history of ulcers, amputations, PVD, and osteomyelitis. The respective results were 46.6% (p<0.001), 71.8% (p<0.001), 12.6% (p=0.001), 19% (p=0.010), and 8% (p<0.001), all of which were more pronounced in the LOPS group. In the group of patients with LOPS, comorbidities were more prevalent. This group exhibited a higher incidence of DR, with 41.4% of patients presenting with NPDR and 13.8% with PDR, in comparison to 40.8% NPDR and 9.9% PDR in the RPS group (p=0.431). Additionally, 95.4% of individuals in the LOPS group had dyslipidemia, slightly higher than the 94.8% in the comparison group (p=0.797).

Hypertension was more prevalent in the LOPS sensation group, with a rate of 97.7% compared to 95.8% in the comparison group (p=0.295). CKD was also more common in the LOPS group, with mild CKD observed in 6.9% of patients, moderate in 28.7%, and severe in 9.2%, compared to 8%, 18.3%, and 6.6%, respectively, in the RPS group (p=0.005). Furthermore, 46.6% of patients with LOPS had CVD, compared to 40% in the comparison group (p=0.189). Lastly, the prevalence of vitamin D deficiency was higher in patients with RPS, at 51.6%, compared to 38.5% in the LOPS group, with a significant p-value of 0.010.

### 3.6 Factors associated with LOPS in the study population

Logistic regression analysis was carried out to identify the factors associated with LOPS (**Table 5**). OR and 95% CI were calculated for several variables including age, smoking status, pedal pulses, footwear, history of ulcers and amputation, CKD, DR, PVD, dyslipidemia, hypertension, CVD, and vitamin D deficiency. Additionally, adjusted OR (AOR) was calculated, accounting for confounding variables such as gender, BMI, HbA1c, and duration of diabetes.

**Table 5:** Logistic regression analysis reporting crude and adjusted odds ratio of factors associated with LOPS (n=174).

Loss of protective sensation				
Variables	OR (95% CI)	P value	Adjusted OR* (95% CI)	P value
Age				
<60 years ³60 years	Reference 0.93 (0.52-1.64)	0.81	- 0.74 (0.40-1.37)	0.34
Smoking status				
Non-smokers Smokers	Reference 38.66 (21.24-70.37)	<0.001	- 38.91 (21.18-71.48)	<0.001
Pedal pulses				
Present Absent	Reference 1.01 (0.62-1.64)	0.95	- 1.03 (0.63-1.7)	0.88
Footwear				
Suitable Unsuitable	Reference 0.72 (0.46-1.12)	0.15	- 0.76 (0.48-1.2)	0.24
History of foot ulcers				
No Yes	Reference 3.35 (2.18-5.14)	<0.001	- 3.35 (2.17-5.17)	<0.001
History of amputation				
No Yes	Reference 3.35 (2.18-5.14)	<0.001	- 3.46 (1.48-8.05)	0.004
Chronic Kidney Disease				
No Mild Moderate Severe	Reference 1.81 (0.77-4.24) 0.64(0.17-2.33) 5.19(1.18-22.7)	0.16 0.50 0.03	- 1.76(0.74-4.17) 0.63(0.17-2.34) 6.04(1.34-26.87)	0.19 0.49 0.01
Diabetic Retinopathy				
None NPDR PDR	Reference 1.38 (0.711-2.68) 0.89 (0.58-1.37)	0.34 0.62	1.3 (0.66-2.56) 0.89 (0.58-1.38)	0.43 0.62
Peripheral Vascular Disease				
No Yes	Reference 2.13 (1.18-3.85)	0.01	- 2.05(0.97-4.30)	0.059
Dyslipidemia				
No Yes	Reference 1.10 (0.44-2.87)	0.79	- 1.03 (0.40-2.68)	0.93
Hypertension				
No Yes	Reference 1.87 (0.56-6.19)	0.30	- 1.90 (0.55-6.48)	0.33
Cardiovascular Disease				
No Yes	Reference 1.31 (0.87-1.96)	0.18	- 1.27 (0.83-1.93)	0.25
Vitamin d deficiency				
No Yes	Reference 0.58 (0.39-0.88)	0.12	- 0.50(0.29-0.87)	0.014

OR, odds ratio, p-value <0.05 indicated statistical significance.

*Adjustments were made for gender, DM duration, glycemic control, and BMI.

Collinearity Diagnostics: To ensure the stability of the multivariable logistic regression model, collinearity among predictors was assessed using the Variance Inflation Factor (VIF). The VIF values ranged from 1.03 to 1.36 for all variables included in the model, indicating no evidence of significant multicollinearity. Following adjustment, smoking status, history of ulcers and amputation, and severe CKD emerged as significant factors associated with LOPS. Smokers [OR = 38.91; 95%CI (21.18, 71.48), p < 0.001] were 38 times more likely to have LOPS. The high odds ratio observed for smoking reflects a strong association and is supported by adequate cell counts, indicating that the result is not due to sparse data bias. Likewise, individuals with a history of ulcers [OR 3.35 (2.17, 5.17), p < 0.001] and amputation [OR 3.46 (1.48, 8.05), p = 0.004] showed a higher likelihood of LOPS. Moreover, severe CKD [OR 6.04 (1.34, 26.87), p = 0.01] was also significantly associated with LOPS.

## 4 Discussion

Peripheral neuropathy is regarded as a troubling complication of diabetes, linked with distressing and painful clinical consequences such as foot ulcers, amputations, and neuropathic pain. The incidence of these complications rises further when patients have co-existing foot deformities and peripheral vascular disease (10). This retrospective study aims to describe the clinical presentation of patients with DPN and to further compare the clinical features of patients with LOPS and RPS in the foot. The final diagnosis of neuropathy typically necessitates employing a gold standard technique such as a NCS (6) or skin punch biopsy (11). Nevertheless, in everyday clinical practice, simpler and more expedient methods are often utilized to evaluate the signs of nerve damage (11), which in this study involved the use of a 10g monofilament.

More than 80% of patients in the study cohort were 60 years and above. This observation aligns with findings from previous study conducted among Emirati patients in Sharjah, which similarly indicated that more than half of the population with DPN fell within the 61–80 age range (12). Additionally, a study conducted in Qatar identified age above 60 years as a significant predictor of DPN. After adjusting for confounding variables, individuals over 60 years of age were found to have a 2.9 fold increased risk of developing DPN compared to younger age groups (p <0.0001) (13).

The distribution of males and females diagnosed with DPN is approximately equal among the population, with a slightly higher prevalence observed in males. While some studies have identified that male sex is a risk factor for DPN (14, 15), others have not demonstrated any significant sex-based predisposition (16–18). The higher prevalence in males may be explained by behavioral and lifestyle differences, including greater engagement in high-stress occupations, higher rates of smoking and alcohol consumption, and lower adherence to treatment regimens compared to females (14).

A notable 91.7% of the study population had a diabetes duration of ≥10 years. A meta-analysis comprising 16 articles reports that 13 studies identified diabetes duration as a significant contributing factor to the development of DPN (10). Furthermore, the American Diabetes Association (ADA) recommends screening for DPN at the time of diagnosis in individuals with T2DM, emphasizing the risk associated with prolonged exposure to hyperglycemia (19).

The cohort demonstrated an approximately equal proportion of smokers and non-smokers. A study in Bahrain found a comparable prevalence of smokers, with 57% of patients with DPN being smokers and having twice the likelihood of developing DPN compared to non-smokers [OR = 2.18 95% CI (1.16,1.77) p=0.002] (12).

The mean height and weight observed in the study population were 161.5cm and 80.3 kg, respectively. This aligns closely with a study conducted among Emirati patients, which reported mean height and weight values of 159.9cm and 80.2kg for DPN patients (20). Although some studies have identified height as a potential predictor of peripheral neuropathy incidence, their conclusions have been inconsistent. This relationship is primarily attributed to the length-dependent nature of the disease. As height increases, so does the length of nerve fibers, thereby increasing the surface area susceptible to potential damage. Consequently, nerve recovery time is prolonged with longer nerve fiber length (20–22). In terms of weight, a meta-analysis demonstrated a significant correlation between the increase in weight and the onset of DPN (p<0.01) (23).

Regarding BMI, this study found that 51.4% of the population were classified as obese. Various research works have indicated a link between overall obesity and the likelihood of developing DPN (24–26). However, research indicates that central obesity, measured by waist circumference may be a stronger predictor of DPN than BMI or other anthropometric measures (27). However, this study lacked a measurement of waist circumference to compare with the literature. Proposed mechanisms explaining how fat contributes to neuropathy include the activation of inflammatory cascades triggered by visceral fat (27). The high obesity rate observed in our study underscores the need for targeted interventions focusing on weight management and modifiable risk factors, regardless of diabetes duration or glycemic control.

The present study cohort demonstrated a high prevalence of co-morbidities, suggesting a strong interrelationship between DPN and other diabetes-related micro- and macrovascular complications. This high prevalence suggests a shared underlying pathogenic mechanism. The current study showed that nearly the entire study population was diagnosed with hypertension. A previous study also showed that after conducting multivariate logistic analysis, hypertension was still a significant predictor of DPN [OR 2.02 (1.77,2.24) p<0.01] (12). The mechanism involves the impact of hypertension on endothelial health, axonal atrophy, and nerve ischemia. Therefore, it is recommended that maintaining optimal glycemic levels in diabetic patients should be complemented by intensive blood pressure management to prevent and delay the onset of DPN.

Dyslipidemia was the second most common comorbidity. Its role as a contributing factor to DPN has been supported by studies demonstrating higher baseline lipid profiles in patients with rapidly progressing neuropathy compared to those with slower progression (p=0.02) (28).

Large-scale trials also support dyslipidemia as a risk factor for DPN (29, 30), with lipid-lowering therapy potentially offering neuroprotection (31, 32). A Korean cohort study further emphasized the role of dyslipidemia, showing associations with low HDL [OR 5.292] and high TG levels [OR 6.129] in incident DPN (33).

Cardiovascular disease (CVD) was also frequently reported, highlighting shared metabolic and vascular pathways between DPN and macrovascular complications. The DIAD study linked DPN symptoms with increased risks of nonfatal myocardial infarction and cardiac death (34), consistent with other literature (35–37). Microvascular complications such as nephropathy and retinopathy were also prevalent. Elevated urinary albumin excretion (UAER) was identified as an independent risk factor for reduced nerve conduction and abnormal monofilament test results (38). Additionally, studies have linked the severity of diabetic retinopathy (DR) to worsening DPN, suggesting mutual screening strategies and interdisciplinary referrals (39).

A larger proportion of the study population were on combination therapy for diabetes management. This includes the use of oral medications combined with insulin or a combination of insulin/oral medications with injectable therapy. This could be explained by several factors related to the population characteristics, such as poorly controlled HbA1c, longer diabetes duration, and the prevalence of multiple co-morbidities. As outlined in the Emirates Diabetes Society (EDS) consensus guidelines for T2DM management, it is recommended to personalize pharmacotherapy based on the individual’s risks, including those related to cardiovascular health, renal function, hypoglycemia, and weight management (40).

The American Association of Clinical Endocrinologists (AACE) and the American College of Endocrinology also recommend starting dual therapy, typically with metformin unless contraindicated or poorly tolerated, along with a second medication, for patients with HbA1c levels exceeding 7.5% (41, 42). Given the average HbA1c level of the study population, it explains the greater use of combination therapy as recommended by these guidelines. Evidence from systematic reviews indicated that pharmacological interventions, such as TCAs and anticonvulsants, are recommended as first-line treatment for managing pDPN, with SNRIs or opioids suggested as second-line therapeutics (43, 44). This strategy is also recommended by the International Association for the Study of Pain (IASP) and the National Institute for Health and Care Excellence (NICE) (45). The treatment used among the study sample indicates the adoption of this approach, as nearly all patients taking pain medications were prescribed anticonvulsants.

Over 91% of patients exhibited DPN symptoms, with positive symptoms (e.g., burning, tingling) being more common. Similar trends were observed in Indian and Saudi studies (46, 47). Absent pedal pulses were found in 22% of participants, lower than the 36.6% seen in German populations (48). However, the prevalence of active ulcers (28%) was substantially higher than in comparable cohorts (40), potentially due to factors like PVD, poor footwear, and recruitment from a secondary care setting. A significantly higher history of ulcers (56%) compared to other studies (e.g., 15%) may be attributed to the longer diabetes duration in this cohort.

Additionally, factors such as the presence of PVD within our population, a high prevalence of inappropriate footwear, and the utilization of data from a secondary care unit (UHS), as opposed to the other study which recruited patients from a quaternary healthcare unit, may predispose patients to foot ulceration. In our study, there was a significantly higher proportion of patients with a history of ulcers compared to the findings reported by Kisozi et al. (49) in a cross-sectional study (56% vs. 15%, respectively). This variance could be explained by differences in the duration of DM, as our study primarily included patients with a duration of ≥10 years of DM, whereas the other study included predominantly newly diagnosed DM patients.

Moreover, a relatively low prevalence of osteomyelitis was noted in the study, contrasting with higher rates reported in other studies (50, 51). This finding is promising, suggesting that within our study population, despite the presence of ulcers, the risk of these ulcers progressing into infections is minimal. It implies that ulcers are being promptly managed and treated in the hospital setting. Additionally, complications such as a history of amputation and PVD were less common in our study population, aligning with findings in existing literature (52, 53). However, the prevalence observed in our study remains higher than what has been reported in the literature. This difference may be attributed to the characteristics of our study population, which predominantly comprises older individuals with longer durations of diabetes and multiple comorbidities.

A considerable proportion of the study participants, amounting to 71%, were observed to be wearing inappropriate footwear, a higher percentage than that reported by Esther et al. (54), who found that 59.4% of patients were using inappropriate footwear. This variation could stem from insufficient education and awareness regarding the importance of wearing suitable footwear among individuals with DPN. Furthermore, 46% of the patients were found to have vitamin D deficiency. In a study investigating the relationship between vitamin D deficiency and DPN, a higher prevalence was noted, with 71.9% of patients exhibiting this deficiency (20). The relatively lower prevalence observed in our study may be attributed to increased awareness of the consequences of vitamin D deficiency on neuropathy and, consequently, increased utilization of supplements.

Regarding biochemical parameters, the average HbA1c level reported in the study was 8.3 ± 2, closely aligning with findings from a comparable study which reported a similar average of 8.2 ± 1.9 (53). As for the lipid profile results, HDL-c and triglyceride levels were similar to those reported in another study (55), however, our study revealed lower levels of total cholesterol and LDL-c. This disparity could be attributed to the high prevalence of dyslipidemia within our study population. Consequently, there may have been a greater utilization of lipid-lowering medications to reduce LDL-c levels, aligning with the recommendations of the ADA and the NICE guidelines, which advocate for the use of moderate-intensity statins for all patients with T2DM over the age of 40 as primary prophylaxis (56, 57). The renal profile findings in our study, including eGFR (77.8 mL/min/1.73m²), creatinine (97.3 μmol/L), and urea levels, were comparable to those reported in other studies (58, 59).

Comparative analysis showed that patients with LOPS had significantly higher rates of smoking, ulcers, amputations, CKD, PVD, and osteomyelitis. Biochemical parameters including HbA1c, urea, creatinine, and TGs were also worse in this group. Interestingly, vitamin D deficiency was more common in the RPS group, potentially due to lower supplement compliance. Higher HbA1c levels (8.6% vs. 8.1%) in the LOPS group align with evidence showing poorer nerve conduction at HbA1c >8.5% (60). The association of LOPS with complications like ulcers and amputations supports prior findings (61, 62), highlighting the progressive nature of DPN and its impact on foot health.

Regression analysis was conducted to identify variables linked to LOPS. After adjusting for confounding factors, smoking status, history of ulcer, history of amputation, and severe CKD, were found smoking to be significantly associated with LOPS. Notably, smokers exhibited a 38-fold increased likelihood of having LOPS (OR: 38.91, 95%CI 21.18, 71.48, p<0.001), which aligns with previous research indicating smoking as a risk factor for DPN. The induction of oxidative stress, systemic inflammation, and endothelial damage by cigarette smoking may contribute to this risk, regardless of diabetes status (12). Importantly, this study is the first to investigate the association between complete LOPS and smoking status, revealing a notably high odds ratio. Systematic review and meta-analysis indicated a strong association between smoking and both the prevalence and incidence of DPN (63). This association is attributed to cigarette smoke serving as a generator of reactive oxygen species, which can induce cellular oxidative stress in the PNS. This oxidative stress may result in cellular damage and apoptosis (63). Furthermore, smoking has been independently linked to oxidative stress, chronic systemic inflammation, and endothelial dysfunction, irrespective of diabetic status. Through these mechanisms, it may potentiate nerve injury alongside metabolic disturbances. In addition, smoking may exert direct neurotoxic effects and contribute to the development of DPN via hypoxemia and microvascular impairment. Comparable to its deleterious impact on large vessels such as the coronary arteries, smoking can also damage smaller vessels, including the vasa nervorum, thereby promoting the onset and progression of DPN (64). Although the magnitude of the odds ratio was high, it should be interpreted cautiously given the retrospective design and potential residual confounding.

Fortunately, smoking is a modifiable risk factor, and healthcare professionals need to emphasize the need to quit smoking and advise patients to attend smoking cessation campaigns.

Additionally, a history of ulcers and amputation was significantly associated with LOPS, likely due to the sequential nature of these complications following LOPS as the primary trigger. This sequence often involves foot deformity, minor trauma, and the development of DFUs, exacerbated by the presence of PVD, which impedes wound healing and may lead to infection and amputation (65). Therefore, patients with a history of ulcers and amputation require strict monitoring and management due to their increased risk of developing a complete absence of sensation, which would predispose them to further complications.

Regarding the association between severe CKD and neuropathy, NICE guidelines highlight diabetic patients with severe renal impairment as high risk for foot complications. Studies have reported a significant increase in the risk of DPN in patients diagnosed with stage 3 and 4 CKD, emphasizing the need for early intervention to address the close association between these microvascular complications (66). The association between severe CKD and LOPS may be explained by several pathophysiological mechanisms. Progressive renal impairment leads to accumulation of uremic toxins, increased oxidative stress, chronic systemic inflammation, and endothelial dysfunction (67, 68). These factors contribute to microvascular injury and impaired perfusion of peripheral nerves. Reduced clearance of metabolic byproducts and heightened inflammatory burden may accelerate axonal degeneration and impair nerve regeneration. Consequently, patients with advanced CKD may experience more rapid progression from mild neuropathy to loss of protective sensation.

This is the first study in the UAE to compare clinical profiles of patients based on 10 g monofilament testing, using a representative sample and excluding non-diabetic causes of peripheral neuropathy. Findings are consistent with international studies, enhancing external validity and clinical relevance (69–71). However, as a retrospective, single-center, cross-sectional analysis, limitations include reliance on medical records, potential selection bias, and inability to infer causality.

## 5 Limitations

This study has several limitations that should be considered when interpreting the findings. First, the retrospective design relies on existing medical records, which may be incomplete or subject to reporting bias, limiting causal inferences. Second, neuropathy assessment was based solely on the 10g monofilament test, without confirmatory nerve conduction studies or skin biopsies, potentially underestimating subclinical neuropathy. Third, the study included only patients with diabetic peripheral neuropathy (DPN), excluding non-diabetic neuropathies, which may limit generalizability. Finally, residual confounding from unmeasured variables (e.g., lifestyle factors, compliance with medications) cannot be entirely ruled out, despite adjustment for known risk factors. Future prospective, multi-center studies with comprehensive diagnostic tools are warranted to validate and extend these findings.

## 6 Conclusion

The findings of this study are consistent with previous research on the clinical characteristics patients with DPN. However, novel associations emerged when comparing patients with varying degrees of protective sensation loss. Patients with LOPS demonstrated a higher prevalence of risk factors such as smoking, abnormal creatinine, reduced eGFR, elevated urea and triglycerides, low HDL-c, active and past foot ulcers, prior amputations, chronic kidney disease (CKD), and osteomyelitis. Further analysis identified smoking, a history of foot ulcers or amputation, and advanced CKD as significant correlates of LOPS sensation. These findings underscore the critical need for early identification and targeted management strategies to mitigate the progression and complications of DPN.

## Data Availability

The original contributions presented in the study are included in the article/supplementary material, Further inquiries can be directed to the corresponding author.
